# Animal-Assisted Interventions With Dogs in Special Education—A Systematic Review

**DOI:** 10.3389/fpsyg.2022.876290

**Published:** 2022-05-31

**Authors:** Jana Meixner, Kurt Kotrschal

**Affiliations:** ^1^Department of Evidence-Based Medicine and Evaluation, University for Continuing Education Krems, Krems an der Donau, Austria; ^2^Department of Behavioral Biology, University of Vienna, Vienna, Austria; ^3^Konrad Lorenz Research Station, Grünau im Almtal, Austria

**Keywords:** animal-assisted interventions, dog-assisted interventions, special education, special pedagogy, human-animal interaction, dogs, *canine* (dog)

## Abstract

Dogs are becoming increasingly popular in pedagogical settings. Particularly children with special educational needs are believed to benefit from dog-assisted interventions. However, reliable evidence for supporting such claims is still scarce and reports on the effectiveness of this approach are often anecdotal. With our review we aim at evaluating the literature to answer the question, whether dog-assisted interventions in an educational setting can help children with special educational needs to improve and to develop their emotional, social and cognitive skills. Following the PRISMA Guidelines, the literature was systematically searched for experimental studies until February 2021. Eighteen studies were finally included, which varied greatly in type of intervention, outcomes measured, sample sizes, and scientific quality, which precluded a formal meta-analysis. Hence, we resorted to a narrative synthesis. Overall, the studies report mixed results in the different functional domains of stress reduction, motivation, social skills, cognitive abilities, reading abilities, social conduct, and mental wellbeing. No study reported any negative effects of the intervention. The most unequivocal evidence comes from studies on dogs’ effects on physiological stress response in challenging situations and on motivation and adherence to instructions, reporting significantly lower levels of cortisol in both children and pedagogues in the presence of dogs, as well as increased motivation to learn and participate. Findings for other outcomes, academic or social, however, remain inconclusive. Data on long-term effects are lacking altogether. Still, this review indicates the potentials of dog-assisted interventions in special pedagogy, particularly towards supporting a calm and trustful social atmosphere.

## Introduction

Human nature seems to come with a specific affinity for nature and other living beings, known as biophilia (Fromm, 1964; Wilson, 1984; Kotrschal, 2019). In fact, the idea that humans enjoy positive effects from living with animals is supported by a number of positive health effects, including cardio-vascular, as well as an increased resilience against mental problems such as anxiety and depression (for reviews see Wells, 2009; Julius et al., 2012; Fine, 2015; Friedman and Krause-Parello, 2018). Hence, biophilia provides a major evolutionary-theoretical basis for research and practice in the field of human-animal relations (Julius et al., 2012; Friedman and Krause-Parello, 2018). Companion animals may satisfy the basic human need for loving and being loved even in a more “essentialized” way as compared to human partners, as they do not judge their human partners looks, wealth, health, intelligence, or political orientation. Furthermore, companion animals may function as social lubricants/catalysts, promoting social contacts between humans (Eddy et al., 1988; Mader et al., 1989; McNicholas and Collis, 2000) and may socially support their human partners by backing and comforting them in demanding situations (Vagnoli et al., 2015; Crossman et al., 2018; Krause-Parello et al., 2018; McCullough et al., 2018).

Children show great interest in animals, and the younger, the greater this interest (Melson, 2009; Wedl and Kotrschal, 2009; DeLoache et al., 2011). As ontogeny may at least coarsely repeat phylogeny, such overwhelming importance of animals at an early age may be considered as a window into the recent evolutionary history of *Homo sapiens* (reviewed in Kotrschal, 2014), pointing at the importance of animals in the evolution of modern humans. Also, by their universal animal orientation, children themselves define contact with animals and nature as a crucial condition for their optimal development (Kotrschal, 2019). Ignoring this may lead to a “nature deficit syndrome” (Louv, 2008; Kotrschal, 2014) and sub-optimal executive functions (Diamond, 2013). Thus, growing up in the company of, and in good relationships with, dogs or other animals seems to support social competence, empathy, cognition and even good health in adults (Melson, 2009; Endenburg and van Lith, 2011).

In fact, dogs are man’s oldest and also socially most compatible and responsive companion animal (Kotrschal, 2014, 2016, 2018a,b). It is therefore not surprising, that they have been playing a central role in animal-assisted interventions since the 1960s, when psychotherapist Boris Levinson (United States) discovered that children opened up when his dog was present during sessions (Levinson, 1965; Podberscek et al., 2005).

Particularly children with special educational needs and/or emotional-behavioral disorders are at high risk of experiencing academic failure and negative feedback, and therefore, develop fearful and aversive attitudes toward school, which in turn block academic and societal success. Such a vicious circle is often started by suboptimal attachment patterns (disorganized or insecure attachment) developed in early childhood (Julius et al., 2012).

In special education, a majority of the children show suboptimal attachment styles and emotional dysregulation, impaired social skills and executive functions are common among children in need of special educational support (Julius et al., 2012). As they often do not find security and social support in other people, a major argument for animal assistance in such pedagogical settings is that the negative internal social working model of suboptimal attachments styles may not be fully transferred to animals (Kurdek, 2008; Wedl et al., 2015). Hence, those children might be particularly responsive to the positive effects of dog-assisted interventions.

By definition, the term animal-assisted intervention covers the overlapping categories animal-assisted therapy, animal-assisted activities and animal-assisted education (Friedman and Krause-Parello, 2018). Animal-assisted therapy usually is highly structured and professionally administered by a trained professional, indicated for a defined population and pursuing a certain clinical goal. In contrast, animal-assisted activities are not necessarily administered by a trained professional, nor in a health care context (Jegatheesan et al., 2014). Following this definition, our review is concerned with animal-assisted activities. However, as it is more commonly used in pedagogy, we used the term animal-assisted interventions instead, but explicitly did not include studies on animal-assisted therapy here.

Promising results suggest that integrating dogs in educational curricula can help children learn and make school more attractive to them (for review see Brelsford et al., 2017). Before all, animal-assisted interventions can support a positive, concentrated atmosphere free of negative stress and fear, which is the precondition for optimal learning (Diamond, 2013; Beetz, 2017). The possible calming and concentration-enhancing effects of animals are probably based on a combination of different interlinked neuronal and mental mechanisms (Wilson, 1984; Kotrschal, 2014, 2019; Carter and Porges, 2016; Beetz, 2017).

Perhaps the most important mechanism is the activation of the oxytocin system (Beetz et al., 2012b; Uvnäs-Moberg et al., 2011; Julius et al., 2012). Oxytocin antagonizes the synthesis of the stress-related steroid hormone cortisol, supports bonding and attachment as well as empathy and positive social behavior. It is released through childbirth, breast feeding, and different kinds of pleasant body contact, be it humans or animals (Nagasawa et al., 2015; Uvnäs-Moberg et al., 2011). Despite being a basis social human need, physical contact between teachers and student is rare and generally discouraged. In contrast, the threshold for bodily contact with animals is much lower, providing an opportunity for unconditional, stress-free and comforting body contact, particularly in cognitively, and socially challenging situations.

In recent decades, animal-assisted activities and interventions in schools, youth welfare and health care facilities developed as worldwide grassroot-movements—not driven by science or theory development, but by positive experience in practice. Scientific evidence toward the effectiveness of animal-assisted activities and interventions was long lagging behind and is catching up only recently (Brelsford et al., 2017; Gee et al., 2017).

Although dog-assisted education has been studied and reviewed in the past (Brelsford et al., 2017), a concise review of the specific effects of animal-assisted interventions on children with special educational needs is lacking so far. This would be particularly useful, as institutions, governmental, and other, need to justify the extra effort and odds potentially associated with animal-assisted interventions. In this review, we tackle the following questions: Is there robust evidence, that dogs can help children with special educational needs to improve their emotional, social, and cognitive skills, academic performance and learning? Can dogs facilitate concentration and motivation and make learning more rewarding for these children?

## Materials and Methods

### Literature Search

This systematic literature review was conducted according to the PRISMA guidelines for systematic reviews and meta-analyses, following a priorly established protocol (Moher et al., 2009). We searched the databases Pubmed, Scopus, Embase, PsycINFO, and Taylor and Francis Online, from their start date to February 2021. We also searched the references of recent systematic reviews on the topic, as well as the *Hochschulschriften* Database of the University of Vienna and the database ProQuest for unpublished dissertations. In accordance with our own language capabilities, we limited searches to English and German language literature.

As search terms we used “special education,” “special pedagogy” and “youth welfare,” “attachment,” “autism” and “adhd” and variations of these terms, and combined them with “animal-assisted” and “dogs” (for the full search strategy and criteria for inclusion, see Supplementary Material).

### Eligibility of Studies

The criteria for eligibility were defined *a priori* following the PICO(TS)-scheme. We included studies that had been conducted in an educational setting involving children and adolescents with special educational needs up to 18 years of age, focused on the effects of animal-assisted interventions incorporating dogs, and investigated outcomes concerning the children’s social or cognitive or academic performance, stress parameters, mental health or subjective wellbeing.

We only included controlled interventional study with no animal-assisted intervention in the control group or control condition, providing quantitative data, or data from which quantitative data could be extracted. Cross-over studies, where participants served as their own control were included as well. No restrictions on study duration were being made.

We excluded studies on incorporating dogs in therapeutic programs or explicit animal-assisted therapy settings. Furthermore, we excluded studies concerned with animal-assisted interventions and physical health outcomes only (e.g., fitness or obesity), case series and case reports without controls, anecdotal reports, cross-sectional studies or surveys and studies providing qualitative data only.

### Data Extraction and Quality Assessment

For all studies included, the following information was extracted in priorly established data extraction forms: Study design and setting, population characteristics (including number of participants, diagnosis, gender, and age), type and duration of intervention, control condition, data on the dogs involved, measured outcomes, tools of measurement, and results.

Quality was assessed for each investigated outcome separately using a modified four-domains-version of *The Risk Of Bias In Non-randomized Studies—of Interventions (ROBINS-I)* assessment tool by the *Cochrane Collaboration* (Sterne et al., 2016) (for details see Supplementary Material).

## Results

**FIGURE 1:**
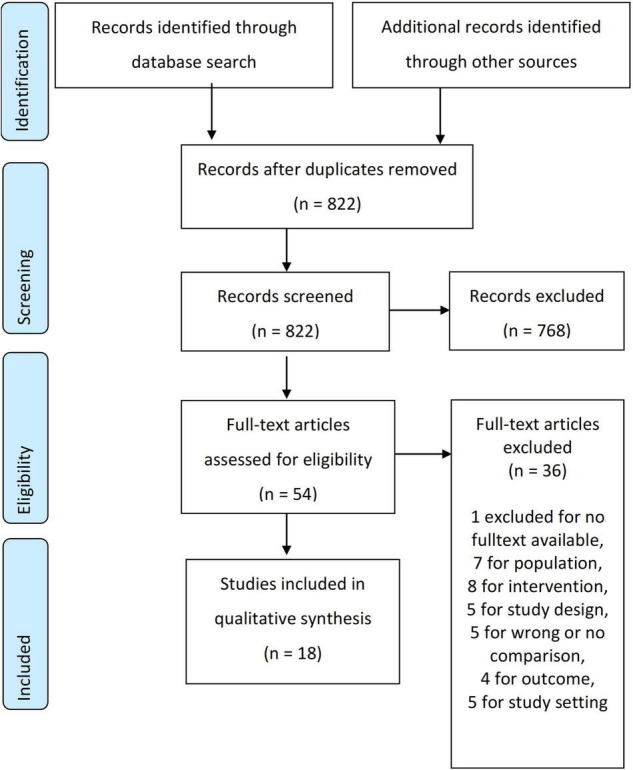
PRISMA flowchart.

We identified 822 records through database searching, of which 54 were eligible for full text screening. Finally, we were able to include 18 studies in the final synthesis (see Figure 1) (for a list of all excluded studies and reasons for exclusion, see Supplementary Material).

Quality, study design and investigated outcome varied greatly between studies. Characteristics of the included studies are summarized in Table 1. Due to the great heterogeneity of the included studies, a meta-analysis of the results was not feasible. Hence, the main results were summarized narratively by outcome.

**TABLE 1:** Characteristics of included studies.

Study	Study design	Country	Population	Intervention	Comparison	Duration	Outcomes (tool used)
Becker et al. (2017)	Controlled trial	United States	*N* = 31 (3 girls, 28 boys); Autism spectrum disorder; 8–14 years	Dog-assisted social skills training (*N* = 17)	Social skills training without dog (*N* = 14)	1 h/week, for 12 weeks	(1) Social skills (SLDT, SRS-2) (2) Theory of Mind (RMET) (3) Depressio*n* (CDI-2)
Beetz et al. (2011)	Randomized controlled trial	Germany/ Austria	*N* = 31 (all boys); Attachment disorders; 7–12 years	Trier social stress test with social support from a friendly dog (*N* = 11)	Trier social stress test with social support from (1) a toy dog (*N* = 9) or (2) a friendly adult (*N* = 11)	1–2 h	(1) Stress response (salivary cortisol) (2) Self-reported stress level (SAM) (3) Interaction with social support (behavior al sampling)
Beetz et al. (2012a)	Randomized controlled trial	Germany/ Austria	*N* = 47 (all boys); Attachment disorders; 7–11 years	Trier social stress test with social support from a friendly dog (*N* = 24)	Trier social stress test with social support from (1) a toy dog (*N* = 13) or (2) a friendly adult (*N* = 10)	1–2 h	(1) Stress response (salivary cortisol) (2) Self-reported stress level (SAM) (3) Interaction with social support (behavior al sampling)
Clune (2019)	Non-randomized controlled trial	United States	*N* = 7 (2 girls, 5 boys); Dyslexia; grade-3 students	Reading to a dog in reading-aloud sessions (*N* = 4)	Reading-aloud sessions without dog (*N* = 3)	2 × 20 min for 7 weeks	(1) Reading fluency (easyCBM) and accuracy (2) Reading anxiety and attitude toward reading (questionnaire)
Esteves and Stokes (2008)	Pre-post study	United States	*N* = 3 (1 girl, 2 boys); Down’s Syndrome, mental retardation; 5–9 years	Play sessions with toys and a real dog	Play sessions with toys including a toy dog	8-min sessions, 5x/week;	Communicative behavior toward dog/caretaker (behavior al sampling)
Gee et al. (2009)	Crossover design	United States	*N* = 11 (5 girls, 6 boys); Mixed sample of an inclusive preschool classroom (5 typically developed, 9 SEN-students); 3–5 years	Gross motor skills tasks performed together with a dog	Gross motor skills tasks performed together with (1) a toy dog, (2) a friendly adult or (3) alone	15–20 min	Adherence to instructions (7-points scale and video recording)
Gee et al. (2010b) Experiment 1	Crossover design	United States	*N* = 12 (6 girls, 6 boys); Mixed sample of an inclusive preschool classroom (5 typically developed, 7 SEN-students); 3–5 years	Object recognition/memory task performed in the presence of a dog	Object recognition/memory task performed in the presence of (1) a toy dog or (2) a friendly adult	NR	Adherence to task (number of prompts needed to perform task)
Gee et al. (2010b) Experiment 2	Crossover design	United States	*N* = 10 (5 girls, 5 boys); Mixed sample of an inclusive preschool classroom (5 typically developed, 5 SEN-students); 3–5 years	Object recognition/memory task performed in the presence of a dog	Object recognition/memory task performed in the presence of (1) a toy dog or (2) a friendly adult	NR	Adherence to task (number of prompts needed to perform task)
Gee et al. (2010a)	Crossover design	United States	*N* = 12 (5 girls, 7 boys); Mixed sample of an inclusive preschool classroom (7 typically developed, 5 SEN-students); 3–5 years	Object categorization task performed in the presence of a dog	Object categorization task performed in the presence of (1) a toy dog or (2) a friendly adult	3 × 10 min within 3 weeks	Accuracy of category choice (correctly identifying the object that “goes with” another object)
Gee et al. (2012b)	Crossover design	United States	*N* = 17 (10 girls, 7 boys); Mixed sample of an inclusive preschool classroom (11 typically developed, 6 SEN-students); 3–5 years	Object categorization task performed in the presence of a dog	Object categorization task performed in the presence of (1) a toy dog or (2) a friendly adult	NR	Accuracy of category choice for animate vs. inanimate objects (correctly identifying the object that “goes with” another object)
Gee et al. (2012a)	Crossover design	United States	*N* = 20 (9 girls, 11 boys); Mixed sample of an inclusive preschool classroom (12 typically developed, 8 SEN-students); 2–5 years	Object recognition/memory task performed with a dog as collaborator	Object recognition/memory task performed with a friendly adult as collaborator	NR	Accuracy and speed of object recognition
Hutter (2015)	Crossover design	Austria	*N* = 9 (4 girls, 5 boys); Attachment disorders, behavior al problems; 5–12 years	Two attachment-based dyadic play sessions with a pedagogue and dog	Two attachment-based dyadic play sessions with a pedagogue alone	4 sessions of 20 min each	(1) Positive and negative social interactions (behavior sampling) (2) Stress response during sessions of children and pedagogues (salivary cortisol)
Kirnan et al. (2020)	Retrospective pre-post study	United States	*N* = 4 (all boys); Learning and behavioral problems; age not reported	Visiting and reading sessions with a dog	Retrospective data collected before intervention started	5–13 sessions from 2013 to 2015	Children’s conduct in the classroom according to teacher-protocols
Le Roux et al. (2014)	Randomized controlled trial	South Africa	*N* = 102 (gender not reported); Students identified as very poor to poor readers; 7–13 years	Sessions of reading to an adult and a dog (*N* = 27)	(1) Sessions of reading to an adult (*N* = 26), (2) reading to a teddy bear (*N* = 24) or 3) no intervention (N = 25)	10 weekly sessions of 20 min each; Re-assessment after 18 weeks	Reading rate, accuracy and comprehension (Neale Analysis of Reading Ability)
Limond et al. (1997)	Crossover design	United Kingdom	*N* = 8 (6 girls, 2 boys); Down’s Syndrome; 7–12 years	7 min interaction with a dog	7 min interaction with a toy dog of same size	14 min (2 × 7) once a week for 6 weeks	Communicative behavior toward dog/caretaker (behavior al sampling)
Martens (2015)	Crossover design	Austria	*N* = 50 (22 girls, 28 boys); Socially challenged children/juveniles with adjustment disorders and/or problems with social conduct or learning in a therapeutic housing program; 5–17 years	Dinner situation in the presence of a friendly dog	Dinner situation without dog	5 visits during dinner (2 with dog, 2 without dog)	(1) Stress level of participants during dinner (salivary cortisol) (2) Behavior and social atmosphere during dinner (behavior sampling)
Somervill et al. (2009)	Crossover design	United States	*N* = 17 (4 girls, 13 boys); Attention deficit/hyperactivity disorder and/or oppositional defiant disorder; 9 years	15-min sessions with 5 min body contact with a dog	15-min sessions without a dog	(1) intervention- and 1 control- sessions on (2) test-days	(1) Systolic and diastolic blood pressure and heart rate (upper arm blood pressure device) (2) Behavior after sessions: mood, attention, anxiety, calmness and disruptive behavior rated by teacher on a 5-point scale
Uccheddu et al. (2019)	Randomized controlled trial	Italy	*N* = 9 (2 girls, 7 boys); Autism spectrum disorder; 6–9 years	Reading sessions in the presence of a dog (*N* = 5)	Reading sessions without a dog (*N* = 4)	10 session of 30 min over a period of 70 days	(1) Reading ability (MT2, MCF, TORC) (2) Cognitive abilities (WISC IV, Vineland Test, IQ)

*N, number of participants; NR, not reported; SEN, special educational needs; SLDT, Social Language Development Test; SRS-2, Social Responsiveness Scale, Second Edition; RMET, Reading the Mind in the Eyes Task; CDI-2, Children’s Depression Inventory, Second Edition; SAM, Self-assessment Manikin; easyCBM, easyCBM Passage Reading Fluency Assessment; MT2, Cornoldi Reading Test; MCF, metaphonological competence; TORC, Test of Reading Comprehension; WISC IV, Wechsler Intelligence Scale for Children; IQ, Intelligence Quotient.*

### Physiological Parameters of Stress

Five studies reported on stress parameters, salivary cortisol or heart rate and blood pressure. In two studies (Beetz et al., 2011, 2012a) the presence of a dog during a standard stress test situation resulted in statistically significant lower overall cortisol levels in boys with attachment disorders (insecure or disorganized attachment style), compared to social support by a friendly human or a toy dog. With the dog, in contrast to the other conditions, cortisol rose only slightly and dropped significantly more rapidly after the test than in the other conditions. Furthermore, there was an inverse relationship between intensity of interaction with the dog initiated by the child and salivary cortisol, with stroking and petting correlating with low cortisol levels and faster drops of cortisol after a stress response.

Hutter (2015) investigated the effects of a dog to attachment-based dyadic play sessions of children with attachment and behavioral problems and their social pedagogues, compared to the same sessions without a dog. Although the children’s cortisol levels remained unrevealing, an effect was found in the in the involved pedagogues: They showed significantly lower cortisol levels in the presence of the dog, which also correlated inversely with the intensity of interaction.

Martens (2015) investigated how the presence of a dog would affect stress levels during communal dinner in a therapeutic housing program for children with adjustment disorders or problems with social conduct or learning. Although remarkable positive effects on behavior, communication and social atmosphere were observed in the presence of the dog, cortisol levels were not different as compared to the same situation without a dog.

Lastly, Somervill et al. (2009) tested the potentially calming effect of brief body contact with a lap dog on children with attention deficit disorder and reported mixed results. The expected overall calming effect could not be confirmed in this study, as they observed increased blood pressure and decreased heart rate in the children after 5 min of holding the small dog on their laps.

### Social Skills/Styles of Interacting and Communicating

Five studies focused on the effects of dog-assisted interventions on social skills and the quality of communication between children and pedagogues.

Hutter (2015) reported that nine children with attachment and behavioral problems showed significantly more positive social behavior and attentiveness toward the pedagogue as well as enhanced play commitment, and less aggressive and obsessive-compulsive behavior, when a dog was involved. Similar positive effects could be observed in the involved pedagogues.

In Martens (2015) the presence of a dog during dinner in a therapeutic housing program resulted in improved communication, featuring overall more talking and involvement in conversation, more cheerful and relaxed behavior and less nervousness and aggressive behavior in the 50 children. In addition, when the dog was present, the atmosphere during dinner was rated as less tense and noisy.

Both Limond et al. (1997) and Esteves and Stokes (2008) looked into the effects of playful interactions with a therapy dog in children with Down’s Syndrome, with the second study intending to replicate the design of the preceding one. Both studies yielded mixed results: Limond et al. (1997) found that the children did not initiate communication with the involved pedagogue more frequently when the dog was present, although the children were significantly more interested in the dog than in the toy dog that was provided in the control condition. In contrast, Esteves and Stokes (2008) found that the children initiated positive interactions with their pedagogues significantly more often when the dog was present. However, no difference in negative social interactions was found between dog and no-dog conditions.

Lastly, Becker et al. (2017) found no statistically significant improvement of emotion recognition as well as verbal and non-verbal social skills after training with a dog in children with autism spectrum disorders, compared to the same training without a dog.

### Motivation, Concentration and Adherence to Tasks

In Gee et al. (2009), children engaged in a motor skills task either alone or together with a trained therapy dog, a friendly college student or a stuffed toy dog. No difference was found between the four conditions regarding the adherence to instructions. In Gee et al. (2010a,b) children needed significantly fewer instructional prompts in a memory task with a real dog as their “collaborator” compared to performing with a toy dog or a friendly college student. According to the authors, this might reflect a motivation-increasing effect by the dog.

Clune (2019) found improved motivation to read and self-perception as a reader in four students with dyslexia after reading sessions in the presence of a dog, compared to three students with dyslexia who completed the same sessions without a dog.

Only a single study (Uccheddu et al., 2019) provided quantitative data on attendance and motivation to go to school. They found that attendance of reading sessions was significantly higher on “dog-days” (100%) than on no-dog-days (75%).

### Cognitive Abilities

We included four studies reporting on the effects of animal-assisted interventions on cognitive abilities such as memory, object categorization and intelligence quotient (IQ). The sample in the three studies by Gee and colleagues (Gee et al., 2010a,2012a,b) included both, typically developed children and children with special educational needs. In a memory task (Gee et al., 2012a) performance was more accurate when the children were collaborating with a dog as compared to a toy dog or a friendly college student. In Gee et al. (2010a), children made significantly fewer mistakes in a categorization task with a dog as collaborator, compared to the presence of a toy dog or a friendly college student. Gee et al. (2012b) reported mixed results when they replicated the same test in a slightly altered way: In the dog-group, they reported an increase in correct categorizations of animate, but not inanimate objects, proposing that the animal’s presence increases the children’s awareness for the animate world or activates social brain mechanisms of anthropomorphization (Urquiza-Haas and Kotrschal, 2015). But overall, no differences in test performance were found between the dog and non-dog groups.

Uccheddu et al. (2019) tested IQ and social skills after reading sessions of nine children with autism spectrum disorder, five children reading in the presence of a dog, four children reading alone. No significant differences in IQ or cognitive skills between the two groups were found.

### Reading Abilities

The three studies on reading abilities yielded mixed results and neither of them investigated long-term effects of dog-interventions.

Clune (2019) conducted a controlled trial with four children with dyslexia reading to a trained therapy dog in bi-weekly reading-aloud sessions, and three dyslexic children completing the same sessions reading to an adult. The author reported on slightly improved fluency and accuracy and less reading-anxiety after 7 weeks in the dog-group. Sample size is very small and no long-term effects were measured. Furthermore we assume a high risk of bias here, as allocation to intervention- and control-group was not random and important information was missing.

Le Roux et al. (2014) conducted a randomized controlled trial involving 102 children in total, all identified as poor readers. Four groups attended weekly reading sessions for 10 weeks. Eight weeks after completion of the intervention the authors found slight, but significantly greater overall reading accuracy and comprehension in children that had read to a dog as compared to children that had read to an adult, a teddy bear or by themselves. However, no quantitative results for overall reading scores are provided and other substantial information is missing too (selective reporting).

Uccheddu et al. (2019) found no significant increase in reading abilities in their randomized controlled trial with nine children with autism spectrum disorder, who completed ten sessions of reading either in the presence of a dog or without a dog. Also, potential long-term effects of the intervention had not been considered.

### Behavior and Conduct

The two studies on children’s behavior and conduct both found no effect of the presence of a dog.

In their retrospective study, Kirnan et al. (2020) investigated the effects of a dog visitation and reading program in four boys with conduct and learning problems. Contrary to the predictions, even more negative and disruptive behavior occurred on days when the dog was present in the classroom as compared to no-dog days. However, due to small sample size and high risk of bias, these results are of limited value.

Likewise, Somervill et al. (2009) found no differences in teacher-rated behavior and mood of 17 children with attention deficit disorder before and after 5 min of body contact with a lap dog.

### Mental Wellbeing

Becker et al. (2017) provided 31 children with autism spectrum disorders with social skills training, half of them in the presence of a dog. After the intervention, depressive symptoms had decreased in both groups, but there was no significant overall difference in social skills or depressive symptoms between groups.

Beetz et al. (2011) assessed mood and perceived levels of stress in 31 boys with attachment disorders during and after a stressful task. Although children who were socially supported by a dog had lower cortisol levels compared to control conditions, self-reported stress did not differ between groups.

## Discussion

To our knowledge, this is the first systematic review of controlled experimental studies on dog-assisted interventions in a special education context. Similar to previous work on animal-assisted interventions in regular school settings (Brelsford et al., 2017), methodology of most of the studies was weak and a fair proportion of the 18 studies included has a high risk of bias, leading to overall inconclusive results.

Although we found indications of positive effects of dog assistance in the various special pedagogical settings, results on the effects of such interventions on behavioral aspects or academic performance of children with special educational needs were rare. Particularly promising was the evidence that dog-assisted interventions may reduce physiological stress parameters and support concentration and motivation and thereby, to some extent also academic performance.

### Cortisol Levels and Stress

Particularly physical contact to a friendly dog is thought to buffer against stress via increasing levels of the cortisol antagonist oxytocin. As measuring oxytocin is not easily accomplished, the oxytocin antagonist cortisol can be measured to indirectly conclude toward oxytocin and to quantify physiological stress levels (e.g., in saliva) (Uvnäs-Moberg et al., 2011). This had been done in all of the included studies focusing on stress levels (Table 1), except for Somervill et al. (2009), who measured heart rate and blood pressure. Overall, the studies indicate a calming effect of the presence of a dog during stressful tasks. In two studies the authors even reported a positive dose-response-relationship: The more contact a child had with a friendly dog, the lower her/his salivary cortisol (Beetz et al., 2011, 2012a). These findings are supported by studies on the positive effects of dog assistance in typically developing children submitted to stressful situations, i.e., in forensic interviews or medical procedures (reviewed in Friedmann et al., 2011).

However, a number of studies, mainly in typically developing children showed no effect of dog involvement on their cortisol levels during stress-inducing tasks (Somervill et al., 2009; Krause-Parello and Gulick, 2015; Martens, 2015; Kertes et al., 2017; Crossman et al., 2018; Kerns et al., 2018; Krause-Parello et al., 2018). While there are some reports on reduced heart rate and blood pressure in both children and adults when supported by a dog, data on potential cortisol reduction remain scarce and are often contradictory (reviewed in Ein et al., 2018).

A number of studies revealed subjectively less stressed children in the presence of a dog, while this was not reflected in physiological parameters, such as heart rate and blood pressure (Somervill et al., 2009; Krause-Parello and Gulick, 2015; Schretzmayer et al., 2017; Krause-Parello et al., 2018). A possible explanation for this divergence could be that positive excitement about interacting with a dog increased blood pressure, heart rate and even cortisol levels. Moreover, some of the study designs ignore the considerable inter-individual variability in both stress response and attitude toward dogs, increasing the likelihood of false-positive results when sample size is low and a group-based, between-subject design instead of a within-subject design is employed. In contrast, Beetz et al. (2011) found cortisol-dampening effects due to contact with dog in a stressful situation, which, however, was not mirrored by the subjective feelings of the children. As most of these children were diagnosed with suboptimal attachment patterns, this mismatch may be caused by low emotion-consciousness, which is common among insecurely attached people (Julius et al., 2012).

Central questions remain unanswered. For example, the relationships between time of exposure, interaction quality and stress reduction remain unclear, as the question whether there is a cumulative effect of repeated animal-contact (Friedmann et al., 2011), i.e., whether a potential beneficial effect would increase with the number total time of exposure or would decrease due to habituation.

Moreover, the “active ingredient” in dog-assisted interventions still needs to be characterized. For example, most study designs do not allow to distinguish whether it is the animal *per se* that is effectively lowering stress, or the distraction the animal provides, for example during medical examinations or other unpleasant procedures. In their study, Barker et al. (2015) tried to control for this distraction-effect in a dog-assisted intervention and found no effect on stress response that exceeded mere distraction. For clarification, studies with appropriate control conditions are needed.

The findings of Beetz et al. (2011), where the amount of physical contact with the dogs scaled inversely with salivary cortisol, support the idea that tactile stimulation might be an important “active ingredient” of dog-assisted interventions. If active interaction and body contact is indeed responsible for the observed stress-reduction, dogs can provide an opportunity for unconditional contact, freed from the complexity and ambiguity inherent in human interactions and without the risk of being rejected—particularly for children with adverse social experiences (Fung, 2017). The idea that indeed, body contact is the active ingredient is supported by Martens (2015) where the mere presence of a dog which was not touched by the children in a dinner situation resulted in much improved social interactions and communication but did not show in salivary cortisol. Again, well-designed studies are needed to further explore the role of tactile stimulation.

Also, the potential calming on the involved pedagogues in dog-assisted interventions is worth a closer look. Pedagogues in special education are regularly confronted with emotionally challenging situations (e.g., Male and May, 1997). As the significantly lower cortisol levels in dog-assisted pedagogues found by Hutter (2015) suggest, pedagogues too may benefit from the presence of a dog in terms of stress reduction, resulting in a significantly improved pedagogue-child communication supporting the major goal of building trust. In essence, the pedagogue-client-dog triangle is socially dynamic and complex but remains far from being understood.

### Motivation

In virtually all of the included studies concerned with motivation and adherence to tasks as the main outcome, children seemed more enthusiastic and concentrated (Gee et al., 2009, 2010a,b; Clune, 2019) and attendance was higher on intervention-days (Uccheddu et al., 2019). Moreover, teachers observed “increased interest and enthusiasm for school in general and reading specifically” after the implementation of a reading-to-a-dog program (Kirnan et al., 2020). Also, indirect hints on motivational effects are common in most of the studies included in this review. These observations are in line with the results of many more studies, not included in this review (e.g., Bassette and Taber-Doughty, 2013; Heyer and Beetz, 2014; Sorin et al., 2015; Stevenson et al., 2015; Schretzmayer et al., 2017; Linder et al., 2018; Noble and Holt, 2018; Schuck et al., 2018; Rousseau and Tardif-Williams, 2019).

Particularly the findings by Gee and colleagues indicate (Gee et al., 2009, 2010a,b), that dogs at school boost intrinsic motivation. With negative academic experiences and frustration being rather common among children in special education, dogs could be a valuable source of supporting intrinsic motivation. Based on our present review, we suggest that the motivational effects—understudied as they are—could be a most valuable aspect of dog-assisted interventions—and that motivation may be an essential outcome variable to support behavioral conduct and academic performance.

### Reading Abilities

Given the great popularity of dog-assisted reading programs, it comes as a surprise that standard quality research on their efficacy is still scarce. Especially in the US various reading programs with dogs were implemented, for example the *Classroom Canines Program* (Sorin et al., 2015), *Sit Stay Read* (Smith, 2009) or *Reading to dogs-programs (R.E.A.D)* (Noble and Holt, 2018). In general education, reading interventions with dogs were found to have overall positive effects, mainly in terms of improved behavior and motivation, as well as by creating a positive reading-environment (for review see Hall et al., 2016). However, the majority of these studies is of moderate scientific quality, anecdotal, non-blinded and only focusing on short-term effects.

Likewise, there is no sound evidence that dog-assisted reading interventions can improve reading skills in children with special educational needs. None of the three included studies (Le Roux et al., 2014; Uccheddu et al., 2019; Clune, 2019) reported on long-term improvements and two of them found no immediate effect either. It must be noted, however, that all three studies report increased motivation to read, more confidence and/or less anxiety in children reading to a dog instead of a human. For children with special educational needs, who often struggle with a history of academic failure and consequently poor self-esteem (Fung, 2017), this is quite an achievement. The dog as an attentive, benevolent listener that is neither criticizing nor judging can take away pressure and ease anxiety. A number of studies in normally developing children reveal mostly positive effects of a dog on reading performance (Hall et al., 2016). It seems that dog-assisted reading interventions bear great potential to improve reading skills and motivation to read, but long-termed studies with adequate sample sizes are needed to substantiate this impression.

### Other Outcomes

Positive effects on performance in a cognitive task were found in children who collaborated with a dog (Gee et al., 2010a,2012a). However, the authors attributed these results rather to increased motivation and concentration due to the presence of the dogs than to a general improvement in cognitive skills. Long term effects of dog-assisted interventions on cognitive abilities were not investigated. Also, whether dogs can increase academic performance of children with special educational needs or lead to better grades remains unclear, as there seem to be not respective studies.

A majority of studies in our review indicate that dogs can act as a social catalyst and “ice breaker” toward a normal communication and social performance (e.g., Guéguen and Ciccotti, 2008). Particularly for children with special educational needs, a dog may induce positive group dynamics by reducing tension and aggression and foster positive and trustful social behavior and communication (Hergovich et al., 2002; Kotrschal and Ortbauer, 2003; Sprinkle, 2008; Hutter, 2015; Martens, 2015; Correale et al., 2017; Lehner, 2017). Children with suboptimal attachment patterns, suffering from negative mental representations of social relationships, find it hard to socially connect with people and tend to replicate the negative social representations they formed in early childhood with any new human social partner (Julius et al., 2013). Those children could gain social support from dogs and see them as their allies in a challenging environment, as demonstrated by Beetz et al. (2011; 2012a). In addition, dogs can make pedagogues and therapists appear in a better light and less threatening or make them be perceived as “outside the complications of normal educational settings” (Friesen, 2010). Via such mechanisms, dogs may support trustful modes of communication.

Furthermore, it has been proposed that children with impaired social and communicative skills (e.g., children with autism spectrum disorders) could profit from the simplicity and clarity inherent in the communication with a dog. The easy-to-read body language and precise commands given to dogs such as “sit” or “stay” might suit them more than the complex mixture of verbal and non-verbal communication patterns between humans and could create a comprehensible speech environment for them (Prothmann et al., 2009; for review see Fung, 2017). This is not supported by our review, however, probably mainly due to the lack of conclusive studies.

### Animal Welfare and Other Important Considerations

Although a majority of children seems curious and excited about dogs, there is a great individual variability in their respective attitudes (Wedl and Kotrschal, 2009). This should be considered when integrating dogs in (special) education. Children who do not want to interact with dogs (e.g., because of fear or for cultural reasons), tend to be excluded from studies on dog-assisted interventions. Although unavoidable, this pre-selection could have led to a distorted picture of the overall study population. This may be one of the reasons why the available studies do not answer the question, if children who do not like to interact with dogs can benefit from dog-assisted interventions and how possible aversions should be dealt with when implementing dogs in a school routine.

It is remarkable that none of the included studies reported injuries or even minor negative effects of the dog-assisted intervention on the children involved. And it seems that “the animals’ side” was well taken care of. Animal welfare was addressed in most of these studies and the dogs’ wellbeing was taken into account in the study designs. Especially when working with children with special educational needs, the possibility of unpredictable or aggressive behavior on the children’s part should always be considered and pedagogues should be prepared to ensure the dogs’ (as well as the children’s) safety at all times. During the intervention, the dog should be monitored closely for signs of stress and get regular breaks (Ng et al., 2015).

### Problems and Limitations

Reviewing the field in question is particularly constrained by the quality of the studies available. In their comprehensive review, Serpell et al. (2017) pointed out the methodological flaws that characterized research on animal-assisted interventions in the past. All of them still apply to the majority of the included studies in this review: small sample size, non-random sample selection and assignment to conditions, inadequacy of control conditions, lack of standardized procedures and researcher expectancy effect severely impair quality and reliability in many of the studies. In addition, due to the nature of the intervention, blinding of both participants and researchers is rarely possible. None of them looked into the long-term effects of animal-assisted interventions. Consequently, there are no standards for the ideal duration, frequency or number of sessions required (Friedman and Krause-Parello, 2018).

Furthermore, expectations of both researchers and the public also gives way to publication bias: Brelsford et al. (2017), for example, noted that most of the studies included in their review that lacked significant positive results originated through gray literature databases. Therefore, serious publication bias in this field of research can be assumed.

## Conclusion

Settings that promote concentration, positive mood and motivation, counteracting fear and stress create a beneficial social and learning environment (Beetz, 2017). Despite the problems with scientific quality, the relevant research suggests that dog assistance is beneficial along these lines via different routes. The presence of a dog can reduce stress, be a source of motivation and create a better social atmosphere in the classroom or within groups. Although a number of studies did not show significant positive effects, no negative effects of dog-assisted interventions were reported either. Unfortunately, small sample size, short duration and methodological flaws constrain the general validity of the results of a limited set of studies that has been conducted on dog-assisted interventions in a special education context.

Although important questions remain unanswered, the studies included in this review, together with the positive experiences reported by the pedagogues in dog-assisted settings, indicate their great potentials. To fathom the possibilities and potential of dog-assisted interventions in special education, well designed and long-termed studies are much needed.

## Author Contributions

JM planned this systematic review, established the protocol, and conducted the literature search. JM conducted study selection, quality assessment, and data extraction, which were supervised and double-checked by KK. JM and KK equally contributed to writing the manuscript. Both authors contributed to the article and approved the submitted version.

## Conflict of Interest

The authors declare that the research was conducted in the absence of any commercial or financial relationships that could be construed as a potential conflict of interest.

## Publisher’s Note

All claims expressed in this article are solely those of the authors and do not necessarily represent those of their affiliated organizations, or those of the publisher, the editors and the reviewers. Any product that may be evaluated in this article, or claim that may be made by its manufacturer, is not guaranteed or endorsed by the publisher.
