# Supplementary material for: Animal-Assisted Interventions With Dogs in Special Education—A Systematic Review
Source: Front Psychol. 2022 May 31;13:876290. doi: 10.3389/fpsyg.2022.876290 (PMC9197485; doi:10.3389/fpsyg.2022.876290)
Supplement: Supplementary file 1 [file Data_Sheet_1.docx]

Supplementary Material

# Search strategy

Supplementary table 1. PICO-TS question

|  | Include | Exclude |
| --- | --- | --- |
| Population | Children with special educational needs (e.g. attachment disorders, ADHD, autism spectrum disorders, learning or behavioural problems) in special education facilities | Adults (>18 years); caregivers/educators of children with special educational needs; interventions with typically developed children without special educational needs; |
| Intervention | Animal-assisted intervention with a dog in a special education setting | Animal-assisted interventions involving other animals than dogs; animal-assisted therapy (e.g. speech therapy, canine assisted psychotherapy); interventions in a non-educational setting (e.g. in hospitals, group therapy sessions); pet dogs; |
| Comparison | No intervention or any other intervention without dog; within-subject-comparison; | No comparison at all; single case studies lacking comparable baseline-data |
| Outcome | Any outcome related to stress, well-being, motivation or academic, communicative, social or cognitive performance | Other outcomes |
| Time | Any duration | - |
| Study design | Published primary experimental studies involving quantitative data; unpublished dissertations or master theses involving experimental studies with quantitative data; | Case series and case reports, cross-sectional studies or anecdotal reports without experimental design, surveys, opinions, qualitative interviews, reviews and meta-analyses; |

## Database search, 7 February 2021

### Embase

('special education*':ab,ti OR 'youth welfare':ab,ti OR 'special pedagog*':ab,ti OR 'developmental disorder':ab,ti OR 'attachment style':ab,ti OR autism:ab,ti OR autistic:ab,ti OR adhd:ab,ti OR 'attention deficit':ab,ti)

AND

(dog:ab,ti OR dogs:ab,ti OR 'canine assisted':ab,ti OR 'canine-assisted':ab,ti OR 'animal assisted':ab,ti)

### Pubmed

"Education, Special"[Mesh] OR special education*[Title/Abstract] OR “youth welfare”[Title/Abstract] OR special pedagog*[Title/Abstract] OR “developmental disorder”[Title/Abstract] OR "attachment style"[Title/Abstract] OR autism[Title/Abstract] OR autistic[Title/Abstract] OR ADHD[Title/Abstract] OR “attention deficit”[Title/Abstract]

AND

Dog[Title/Abstract] OR dogs[Title/Abstract] OR “animal assisted”[Title/Abstract] OR animal-assisted[Title/Abstract]

### Scopus

TITLE-ABS-KEY("special education*" OR "youth welfare" OR "special pedagog*" OR "developmental disorder*" OR "attachment style*" OR autism OR autistic OR ADHD OR "attention deficit")

AND

TITLE-ABS-KEY(dog OR canine OR "animal assisted")

### PsycINFO

#### Search in abstracts

"special education*" OR “youth welfare” OR "special pedagog*" OR “developmental disorder*” OR "attachment style" OR autism OR autistic OR ADHD OR “attention deficit”

AND

dogs OR animal-assisted OR canine

#### Search in keywords

"special education*" OR “youth welfare” OR "special pedagog*" OR “developmental disorder*” OR "attachment style" OR autism OR autistic OR ADHD OR “attention deficit”

AND

dogs OR "animal-assisted intervention"

# Quality assessment

Supplementary table 2. Quality assessment domains

| Domain | Bias due to |
| --- | --- |
| Selection | - recruitment - unbalanced baseline characteristics - randomization process |
| Comparability | - differences in intervention- and control-conditions |
| Data collection | - lack of blinding of researchers and/or participants - inappropriate methods or measurements |
| Attrition | - high drop-out rate - selective reporting - missing data |

## Quality assessment results

Supplementary Figure 1. Risk of bias assessment of included studies. RoB: Risk of bias; Green: low risk of bias; yellow: moderate risk of bias; red: high risk of bias; grey: no information or not applicable; Martens 2015^1^: Outcome behavior/social atmosphere; Martens 2015^2^: Outcome cortisol

# Studies excluded after full text screening (reason for exclusion)

Albasha, H., Kelly, M., Andrews, J., & Rice, S. (2016). The effects of animal assisted intervention on the social initiation behaviors of children with an autism spectrum disorder. Journal of Investigative Medicine, 64(1), 264. (no fulltext available)

Alison, C. E. (2011). Using dogs in a home-based intervention with children with autism spectrum disorders. (72), ProQuest Information & Learning (wrong setting)

Anderson, K. L., & Olson, M. R. (2006). The value of a dog in a classroom of children with severe emotional disorders. Anthrozoos, 19(1), 35-49. (wrong study design, qualitative)

Avila-Alvarez, A., Alonso-Bidegain, M., De-Rosende-Celeiro, I., Vizcaino-Cela, M., Larraneta-Alcalde, L., & Torres-Tobio, G. (2020). Improving social participation of children with autism spectrum disorder: Pilot testing of an early animal-assisted intervention in Spain. Health Soc Care Community. (wrong setting)

Bassette, L. A., & Taber-Doughty, T. (2013, June). The effects of a dog reading visitation program on academic engagement behavior in three elementary students with emotional and behavioral disabilities: A single case design. In Child & Youth Care Forum (Vol. 42, No. 3, pp. 239-256). Springer US. (wrong or no comparison)

Brandes, H. (2018). The potential of green care interventions to promote positive youth development with a one health lens. (79), ProQuest Information & Learning, (wrong study design)

Connell, C. G., Tepper, D. L., Landry, O., & Bennett, P. C. (2019). Dogs in Schools: The Impact of Specific Human–Dog Interactions on Reading Ability in Children Aged 6 to 8 Years. Anthrozoos, 32(3), 347-360. (wrong population)

Correale, C., Crescimbene, L., Borgi, M., & Cirulli, F. (2017). Development of a Dog-Assisted Activity Program in an Elementary Classroom. Veterinary sciences, 4(4), 62. (wrong population)

Crossman, M. K., Kazdin, A. E., Matijczak, A., Kitt, E. R., & Santos, L. R. (2018). The Influence of Interactions with Dogs on Affect, Anxiety, and Arousal in Children. Journal of Clinical Child & Adolescent Psychology, 1-14. (wrong population)

Fung, S. C. (2019). Effect of a canine-assisted read aloud intervention on reading ability and physiological response: A pilot study. Animals, 9(8), 474. (wrong population)

Gee, N. R., Harris, S. L., & Johnson, K. L. (2007). The Role of Therapy Dogs in Speed and Accuracy to Complete Motor Skills Tasks for Preschool Children. Anthrozoos, 20(4), 375-386. (wrong outcome)

Geldhof, G. J., Flynn, E., Olsen, S. G., Mueller, M. K., Gandenberger, J., Witzel, D. D., & Morris, K. N. (2021). Emotion regulation and specificity: The impact of animal-assisted interventions on classroom behavior. Journal of Applied Developmental Psychology, 73, 101253. (wrong intervention)

Germone, M. M., Gabriels, R. L., Guérin, N. A., Pan, Z., Banks, T., & O'Haire, M. E. (2019). Animal-assisted activity improves social behaviors in psychiatrically hospitalized youth with autism. Autism, 23(7), 1740-1751. (wrong setting, wrong population)

Griffioen, R. E., van der Steen, S., Verheggen, T., Enders-Slegers, M. J., & Cox, R. (2020). Changes in behavioural synchrony during dog-assisted therapy for children with autism spectrum disorder and children with Down syndrome. J Appl Res Intellect Disabil, 33(3), 398-408. (wrong intervention, AAT)

Grigore, A. A., & Rusu, A. S. (2014). Interaction with a therapy dog enhances the effects of social story method in autistic children. Society and Animals, 22(3), 241-261. (wrong intervention, AAT)

Jorgenson, C. D., Clay, C. J., & Kahng, S. (2019). Evaluating preference for and reinforcing efficacy of a therapy dog to increase verbal statements. J Appl Behav Anal. (wrong intervention, AAT)

Juríčková, V., Bozděchová, A., Machová, K., & Vadroňová, M. (2020). Effect of Animal Assisted Education with a Dog Within Children with ADHD in the Classroom: A Case Study. Child and Adolescent Social Work Journal, 37(6), 677-684. (no comparison)

Kirnan, J., Siminerio, S., & Wong, Z. (2016). The Impact of a Therapy Dog Program on Children’s Reading Skills and Attitudes toward Reading. Early Childhood Education Journal, 44(6), 637-651. (wrong population)

Michelotto, A. L. L., Anater, A., Guebert, M. C. C., Borges, T. D., Michelotto, P. V., Jr., & Pimpao, C. T. (2019). Animal-Assisted Activity for Children with Autism Spectrum Disorder: Parents' and Therapists' Perception. J Altern Complement Med, 25(9), 928-929. (wrong study design, qualitative)

Noble, O., & Holt, N. (2018). A study into the impact of the Reading Education Assistance Dogs scheme on reading engagement and motivation to read among Early Years Foundation-Stage children. Education 3-13, 46(3), 277-290. (wrong study design, qualitative)

Obrusnikova, I., Bibik, J. M., Cavalier, A. R., & Manley, K. (2012). Integrating Therapy Dog Teams in a Physical Activity Program for Children with Autism Spectrum Disorders. Journal of Physical Education, Recreation & Dance, 83(6), 37-48. (wrong study design)

O'Haire M, E., McKenzie, S. J., McCune, S., & Slaughter, V. (2014). Effects of classroom animal-assisted activities on social functioning in children with autism spectrum disorder. Journal of Alternative and Complementary Medicine, 20(3), 162-168. (wrong intervention, guinea pigs)

Prothmann, A., Christine, E., & Sascha, P. (2009). Preference for, and responsiveness to, people, dogs and objects in children with autism. Anthrozoos, 22(2), 161-171. (wrong outcome, preference test)

Protopopova, A., Matter, A. L., Harris, B. N., Wiskow, K. M., & Donaldson, J. M. (2020). Comparison of contingent and noncontingent access to therapy dogs during academic tasks in children with autism spectrum disorder. J Appl Behav Anal, 53(2), 811-834. (wrong setting, no educational setting)

Rousseau, C. X., & Tardif-Williams, C. Y. (2019). Turning the Page for Spot: The Potential of Therapy Dogs to Support Reading Motivation Among Young Children. Anthrozoos, 32(5), 665-677. (wrong population)

Schuck, S. E. B., Johnson, H. L., Abdullah, M. M., Stehli, A., Fine, A. H., & Lakes, K. D. (2018). The Role of Animal Assisted Intervention on Improving Self-Esteem in Children With Attention Deficit/Hyperactivity Disorder. Front Pediatr, 6, 300. (wrong intervention, AAT)

Schuck, S. E., Emmerson, N. A., Fine, A. H., & Lakes, K. D. (2015). Canine-assisted therapy for children with ADHD: preliminary findings from the positive assertive cooperative kids study. Journal of attention disorders, 19(2), 125-137. (wrong intervention, AAT)

Silva, K., Lima, M., Fafiaes, C., Sinval, J., & de Sousa, L. (2020). Preliminary Test of the Potential of Contact With Dogs to Elicit Spontaneous Imitation in Children and Adults With Severe Autism Spectrum Disorder. Am J Occup Ther, 74(1) (wrong setting)

Silva, K., Lima, M., Santos-Magalhaes, A., Fafiaes, C., & de Sousa, L. (2018). Can Dogs Assist Children with Severe Autism Spectrum Disorder in Complying with Challenging Demands? An Exploratory Experiment with a Live and a Robotic Dog. J Altern Complement Med, 24(3), 238-242. (wrong setting)

Silva, K., Lima, M., Santos-Magalhães, A., Fafiães, C., & de Sousa, L. (2019). Living and Robotic Dogs as Elicitors of Social Communication Behavior and Regulated Emotional Responding in Individuals with Autism and Severe Language Delay: A Preliminary Comparative Study. Anthrozoös, 32(1), 23-33. (wrong setting)

Sorin, R., Brooks, T., & Lloyd, J. (2015). The impact of the Classroom Canines program on children's reading, social and emotional skills and motivation to attend school. The International Journal of Literacies, 22, 23-35. (wrong or no comparison)

Stevenson, K., Jarred, S., Hinchcliffe, V., & Roberts, K. (2015). Can a dog be used as a motivator to develop social interaction and engagement with teachers for students with autism? Support for Learning, 30(4), 341-363. (wrong or no comparison)

Wedl, M., Kotrschal, K., Julius, H., & Beetz, A. (2015). Children with Avoidant or Disorganized Attachment Relate Differently to a Dog and to Humans During a Socially Stressful Situation. Anthrozoos, 28(4), 601-610. (wrong outcome)

Welsh, K. C. (2010). The use of dogs to impact joint attention in children with autism spectrum disorders. (70), ProQuest Information & Learning (wrong outcome)

Wright Hempel, A. (2007). The effects of animal-assisted therapy on the development of positive interaction skills within the cross-categorical special education population. (67), ProQuest Information & Learning (wrong or no comparison)

Zasloff, R. L., Hart, L. A., & Weiss, J. M. (2003). Dog training as a violence prevention tool for at-risk adolescents. Anthrozoos, 16(4), 352-359. (wrong intervention)
